# Physiological determinants and energy system contribution in short- and middle-distance cycling performance

**DOI:** 10.1007/s00421-025-06049-w

**Published:** 2025-12-19

**Authors:** Lars Erik Gjerløw, Eva Maria Støa, Jan Helgerud, Jan-Michael Johansen, Øyvind Støren

**Affiliations:** 1 Department of Sports, Physical Education and Outdoor Studies, University of South-Eastern Norway, Bø, Norway; 2 Department of Natural Sciences and Environmental Health, University of South-Eastern Norway, Bø, Norway; 3 Department of Circulation and Medical Imaging, Norwegian University of Science and Technology, Trondheim, Norway; 4 Treningsklinikken, Medical Rehabilitation Clinic, Trondheim, Norway

**Keywords:** Aerobic capacity, Anaerobic capacity, Middle-distance performance, Maximal accumulated oxygen deficit, Anaerobic sprint reserve

## Abstract

**Purpose:**

The purpose of this study was to investigate the contribution of aerobic and anaerobic energy release on cycling performance in 10 to 180 s.

**Methods:**

18 male cyclists from recreational- to elite level participated in this cross-sectional study. The cyclists were tested in oxygen cost of cycling (C), peak oxygen uptake (VO_2peak_), maximal sprint power (MSP), and five short time trials (TT): 10 s (10TT), 30 s (30TT), 60 s (60TT), 120 s (120TT) and 180 s (180TT). In all time trials, VO_2_, maximal accumulated oxygen deficit (MAOD) and blood lactate concentration ([La^−^]_b_) were measured.

**Results:**

On average, there was a 50–50% distribution of anaerobic and aerobic energy release at 73 s and at 180TT the distribution was 71% aerobic and 29% anaerobic. When performance was expressed in absolute watt values, strong correlations were found between 180TT and MSP (*r* = 0.85, *p* < 0.01, 95% CI [0.55, 0.93]), maximal aerobic power (MAP) (*r* = 0.88, *p* < 0.01, 95% CI [0.69, 0.95]), MAOD expressed as mL· kg^− 1^ (*r* = 0.81, *p* < 0.01, 95% CI [0.56, 0.93]) and 0.7MAP + 0.3MSP (*r* = 0.91, *p* < 0.01, 95% CI [0.77, 0.97]). MAOD measured in 180TT correlated significantly with MAOD in all the other TTs, i.e., those with the highest MAOD already at 10 s, also had the highest MAOD at 180s. Furthermore, MAOD increased rapidly between 10 and 60 s, but leveled off between 120 and 180 s. This suggests an individual maximal volume of anaerobic work that needs to be distributed across a given cycling duration.

**Supplementary Information:**

The online version contains supplementary material available at 10.1007/s00421-025-06049-w.

## Introduction

Supra-maximal intensity relative to maximal aerobic power (MAP), achieved during short races at approximately three minutes, requires significant contributions from both aerobic and anaerobic energy systems (Spencer and Gastin 2001; Sandford and Stellingwerff 2019; Sandford et al., 2019^a^). Previous studies reported that the aerobic and anaerobic contribution were estimated to be 50/50 after approximately 1 min of cycling with maximal effort, and 20/80 and 75/25 at 30 s and 3.5 min, respectively (Medbø and Tabata 1989; Craig and Norton 2001; Gastin 2001). Middle-distance performance in running and cross-country sprint skiing has previously been shown to be determined by physiological variables such as MAP or the equivalent maximal aerobic speed (MAS), maximal sprint power (MSP) or the equivalent maximal sprint speed (MSS), maximal accumulated oxygen deficit (MAOD), anaerobic power reserve (APR) or the equivalent anaerobic sprint reserve (ASR), and the capacity for lactate production, accumulation, exchange and removal (Ramsbottom at al., 1994; Stöggl et al. 2007; Ingham et al. 2008; Losnegard et al. 2012; Sandford et al., 2019^a^; Bellinger et al. 2021; Støren et al. 2021; 2023; Hallam et al. 2022; Jimenez-Reyes et al. 2022; Watanabe et al. 2024). To avoid confusion or misinterpretation in this study related to the use of abbreviations, power is used as the unit of measurement instead of speed where applicable, for example in MAP, MSP, and APR.

To our knowledge, only a few studies have investigated the impact of both aerobic endurance capacity, maximal sprint ability and anaerobic endurance capacity on short time trial (TT) performance in cycling. Støa et al. (2025) examined the relationship between several physiological variables and 3000 m cycling time trial (mean race time of 158.2 ± 16.8 s). The results showed significant correlations between time performance and MAP and MSP, but not with MAOD or time to exhaustion (TTE). Craig et al. (1993), who investigated physiological determinants in 4000 m individual pursuit in track cycling, found significant correlations between time performance and peak oxygen uptake (VO_2peak_), power output at LT, and with MAOD measured in a 5-minute supra-maximal test. MAP represents the quotient of VO_2peak_ divided by oxygen cost of cycling (C), and has previously been shown to be an important determinant of performance in cycling (Faria et al. 2005; Støren et al. 2013; Babault et al. 2018), middle-distance running (Ingham et al. 2008; Sandford et al., 2019^a^; Bellinger et al. 2021), and cross-country sprint skiing (Stöggl et al. 2007; Støren et al. 2023; Gjerløw et al. 2025). MSP is also a significant variable for performance in typical middle-distance events across several sports (Bentley et al. 1998; Stöggl et al. 2015; Sandford et al., 2019^a^; Jimenez-Reyes et al. 2022; Støren et al. 2021; 2023; Støa et al. 2025). To find the MSP in cycling, measures of either peak or mean power outputs during short all-out tests (< 10 s) are used (Driss and Vandewalle 2013).

MAOD or TTE at a supra-maximal intensity relative to MAP, are often used as measures to quantify the ability to sustain anaerobic metabolism over a given period, often referred to as anaerobic capacity or anaerobic endurance capacity (Medbø and Tabata 1989; Blondel et al. 2001; Noordhof et al. 2011). Accumulated oxygen deficit (AOD) is calculated as the difference between the oxygen demand of a given work and the measured oxygen expenditure used to cover this demand (Medbø and Tabata 1989; Saunders et al., 2004; Hill and Vingren 2011). The possible relationship between either TTE at a supramaximal intensity, or MAOD and time performance in middle-distance events have produced equivocal findings. Negative correlations between MAOD and time performance were found in Craig et al. (1993) for track cycling, Ramsbottom at al. (1994) and Billat et al. (2009) for 800 m running and in Losnegaard et al. (2012) for 600 m treadmill roller skiing. These results are in contrast with other studies not finding TTE and/or MAOD to be associated with middle-distance performance in running (Craig and Morgan 1998; Støren et at., 2021), cross-country sprint skiing (Støren et al. 2023; Gjerløw et al. 2025) and short time trial cycling (Støa et al. 2025).

The relationship between MAOD and MSP has previously been discussed. Støren et al. (2023) suggested that MAOD represents a set individual volume and is limited by the maximal capacity to produce energy through anaerobic energy systems. This suggestion was supported in the results presented in Hill and Vingren (2011), which measured MAOD at 3 min, 5 min, and 7 min time trials in running and cycling and found no significant difference in total MAOD between the different work periods. Theoretically, MSP could then represent an athlete’s maximal anaerobic capacity and thus the upper limit of MAOD. Støren et al. (2023) found a positive correlation between MAOD and anaerobic power reserve (APR). They proposed that MAOD is a result of APR, which is determined by the difference between MAP and MSP. Gastin et al. (1994) found that a group of sprint-trained cyclists produced both higher peak power output and MAOD (in absolute values) in a 90s all-out cycling time trial than a group of endurance trained cyclists. Higher MAOD was also related to performance in specialized cross-country skiing sprinters, compared to distance specialized skiers, in the study of Losnegard and Hallén (2014).

Knowledge about to what extent anaerobic energy release affects short-time endurance performance, and what determines the magnitude of this energy release in different individuals, is of relevance when designing optimal training programs for different athletes. The main purpose of this study was therefore to investigate the contribution of aerobic- and anaerobic energy release, and possible relationships between physiological variables including MAP, MSP and MAOD and performance in several short-time ergometer cycling time trials from 10 s to 180 s. Special emphasis was put on performance in the 180 s, in order to compare cycling results with previous studies done on running and cross-country skiing. The hypotheses were that maximal work would be predominantly covered by anaerobic energy release up to 60–90 s, and that those with the highest MAOD in the shortest trials would also have the highest MAOD in the longer trials, and that MAP would be increasingly important with increased trial duration.

## Methods

### Experimental approach

A cross-sectional study was conducted to explore relationships between physiological variables and performance in short-time cycling time trials from 10 s to 180 s. The variables measured in this study were mean and peak power output in five different short time trials (10 s, 30 s, 60 s, 120 s and 180 s), C, VO_2peak_, MSP, MAOD and blood lactate concentration [La^−^]_b_^−^.

### Subjects

18 male cyclists from recreational- to elite level volunteered to participate in this study (Table 1). The heterogeneity in performance level was deliberately chosen to be able to detect potential relationships between the physiological variables tested and time performance in the different time trials performed.

**Table 1:** Subject characteristics and results (*N* = 18)

Age (years)	32 ± 9	28.1
BM (kg)	85 ± 12	13.6
Height (cm)	184 ± 6	3.4
VO_2peak_		
L·min^− 1^	4.93 ± 0.56	11.3
mL·kg^− 1^·min^− 1^	58.6 ± 8.7	14.8
mL·kg^− 0.67^·min^− 1^	252.6 ± 31.9	12.6
HR (BPM)	185 ± 12	6.6
[La^−^]_b_ (mM)	13.7 ± 3.6	26.3
RER (VCO_2_ / VO_2_)	1.17 ± 0.07	6.0
C		
mL·W^− 1^	14.6 ± 0.7	4.9
mL·kg^− 1^·W^− 1^	0.174 ± 0.020	11.6
mL·kg^− 0.67^·W^− 1^	0.748 ± 0.058	7.7
MAP (W)	337.6 ± 36.7	10.9
MSP (W)	957.0 ± 153.0	16.0
10TT		
W	957 ± 153^*^	16.0
W·kg^− 1^	11.3 ± 1.9^*^	16.8
MAOD (mL·kg^− 1^·min^− 1^)	136.9 ± 24.0^*^	17.5
MAOD (mL·kg^− 1^)	22.8 ± 4.0^*^	17.6
[La^−^]_b_ (mM)	7.6 ± 2.2^*^	28.4
30TT		
W	796 ± 123^*^	15.5
W·kg^− 1^	9.4 ± 1.5^*^	15.9
MAOD (mL·kg^− 1^·min^− 1^)	98.0 ± 20.6^*^	21.0
MAOD (mL·kg^− 1^)	49.0 ± 10.3^*^	21.0
[La^−^]_b_ (mM)	11.3 ± 2.1^*^	18.7
60TT		
W	615 ± 79^*^	12.9
W·kg^− 1^	7.3 ± 1.0^*^	13.4
MAOD (mL·kg^− 1^·min^− 1^)	62.6 ± 10.3^*^	16.4
MAOD (mL·kg^− 1^)	62.6 ± 10.3^**§**^	16.4
[La^−^]_b_ (mM)	14.2 ± 2.8 ^**§**^	20.1
120TT		
W	473 ± 59^**#**^	12.5
W·kg^− 1^	5.6 ± 0.8^**#**^	14.6
MAOD (mL·kg^− 1^·min^− 1^)	34.2 ± 6.4^**#**^	18.6
MAOD (mL·kg^− 1^)	68.4 ± 12.7^**§**^	18.6
[La^−^]_b_ (mM)	14.5 ± 3.1^**§**^	21.4
180TT		
W	422 ± 56^**#**^	13.2
W·kg^− 1^	5.0 ± 0.8^**#**^	15.7
MAOD (mL·kg^− 1^·min^− 1^)	23.4 ± 5.1^**#**^	22.0
MAOD (mL·kg^− 1^)	70.1 ± 15.4^**§**^	21.9
[La^−^]_b_ (mM)	14.5 ± 2.5^**§**^	17.5

Values are mean ± standard deviation, with coefficient of variance in per cent in the right column. BM, body mass. kg, kilogram. VO_2peak_, peak oxygen consumption. C, oxygen cost of cycling. HR, heart rate. BPM, beats per minute. RER, respiratory exchange ratio. W, watts. MAP, maximal aerobic power (VO_2peak_ / C). MSP, maximal sprint power. [La^−^]_b_, blood lactate concentration. mM, millimole per litre. MAOD, maximal accumulated oxygen deficiency

* *p* < 0.01 different from all the other TTs, except *p* < 0.05 for [La^−^]_b_ (mM) between TT30 and TT60

# *p* < 0.01 different from TT10, TT30 and TT60

§ *p* < 0.01 different from TT10 and TT30, except *p* < 0.05 for [La^−^]_b_ (mM) between TT30 and TT60

The study was approved by the institutional research board at the University of South-Eastern Norway, the Norwegian Agency for Shared Services in Education and Research (SIKT, ref. 183455) and conducted in accordance with the Helsinki declaration. All subjects gave their written informed consent to participate after having received oral and written information about the study.

### Testing procedures

The participants in the present study were tested over three different days at the same laboratory, with at least one day in between and within two weeks of the first test. All tests were performed on a test ergometer cycle (Lode Excalibur Sport; Lode, Groningen, The Netherlands), modified with racing pedals and racing handlebars, and calibrated for power output and cadence. Calibration tolerance was set to 1.5% by the manufacturer. Both the handlebars as well as the seat pin were horizontal and vertically adjustable. The riding position was adjusted manually for each participant before the testing started. The ergometer cycle was programmed with a software (Lode Ergometry Manager 10; Lode, Groningen, The Netherlands) designed for precise monitoring of different variables, which was used to adjust braking wheel resistance to the flywheel and to measure mean and peak power output in the different tests performed.

On the first test day, the cyclists performed three different tests: measurement of C, test of VO_2peak_ and a 10 s all-out sprint test. After a submaximal warm-up of 15 min, the C-measurement was carried out as a 5-minute work period at a work intensity corresponding to 70–90% of VO_2peak_. The work intensity was estimated for each athlete. If, after the VO_2peak_ test, the intensity for the submaximal work should be outside the 70–90% area, the submaximal test would be repeated the next test day. However, this was not necessary for any of the cyclists. The average of five VO_2_ measurements between minutes 3 and 5 (3:30, 3:50, 4:10, 4:30 and 4:50) were used to calculate C as VO_2_-expenditure expressed in mL×kg^− 1^×W^− 1^. To ensure steady state, measurements of VO_2_ were controlled for no further increase after three minutes of the test. All VO_2_ measurements were performed with the metabolic test system, Jaeger Vyntus CPX (CareFusion, GmbH, Hoechberg, Germany), with a mixing chamber. The margin of error was set by the manufacturer to 3%, although test-retest measurements at this lab have shown the variation to be less than 1%. After a 5-minute break, a VO_2peak−_test was performed using an incremental protocol. The subjects started at an intensity predicted to represent approximately 70% of peak heart rate (HR_peak_). Every 30 s, the power was increased by 5–25 W based on the subjective evaluation of the test leader. The testing team consistently comprised 2–3 members, with the same test leader present throughout all testing sessions. In addition to voluntary fatigue, heart rate (HR) ³ 95% of HR_peak_, respiratory exchange ratio (RER) ³ 1.05, as well as a plateau of the VO_2_ curve were used as criteria to evaluate if VO_2peak_ was obtained. The mean of the two subsequent highest registered VO_2_-values, each representing 20-seconds intervals by the mixing chamber, was set as VO_2peak_. All HR measurements were obtained using Polar s610 HR monitors (Polar Oy, Kempele, Finland). Based on the VO_2peak_ and C measurements, MAP was calculated as VO_2peak_ × C^− 1^.

After a 30-minute break, the 10 s sprint (10TT) was performed. The subjects were instructed to pedal at a power output of 100 W the last minute prior to the test-start, with a self-managed increase in cadence the last 10 s. Following a countdown of the last 10 s of the initial minute, braking resistance in Nm·kg^− 1^ body mass was automatically applied to the flywheel. The braking resistance was set before the start of each time trial and was not adjustable during the tests. Braking resistance was individually adapted for each participant for the different time trials. For examples, in 10TT the braking resistance factor ranged from 0.8 to 1.1 Nm·kg^− 1^. Immediately after the test was completed, a capillary blood sample to measure [La^−^]_b_^−^ was taken. [La^−^]_b_^−^ was measured with a Lactate Scout+ (SensLab GmbH, Leipzig, ray Inc., Kyoto, Japan). HR_peak_ was also measured in all time trials. The MSP was assessed from the mean power output measurement conducted during the 10TT.

On test day two and three all cyclists performed the four remaining time trials. 30 s (30TT) and 180 s (180TT) were performed on test day two, while 60 s (60TT) and 120 s (120TT) were performed on test day three. On both test days, a sub-maximal warm-up of 15–30 min was performed before the first test, and with a 30-minute break between tests. The one-minute pre-test routine, previously described for 10TT, was identical in all time trials. The braking wheel resistance was individually customized for all the participants based on the duration of the test and individual preference for cadence during each test.

Based on prior studies with the same maximal work for the same time duration (Støren et al. 2021, 2023; Gjerløw et al. 2025), we used the Eq. 0.7MAP + 0.3MSP in order to investigate the correlation between this equation and 180TT performance.

In all time trials, MAOD was calculated as the mean difference between the mean VO_2_-demand and the mean measured VO_2_ expenditure. This method was also used in recent publications with similar protocols, i.e., Støren et al. (2023) and Støa et al. (2025). In this study, we also used corrected maximal accumulated oxygen deficit values (MAODcorr). In short, MAODcorr accounts for the oxygen stored in venous blood, which is utilized during muscle work, in the estimation of oxygen consumption. The stored oxygen is used because there is a delay between the increased oxygen demand, and consequently, the oxygen uptake in the muscles during intensified work, and the corresponding rise in oxygen intake through inspired air to sufficiently oxygenate the blood.

**Table:** 

Methods box: example of MAOD computation; uncorrected and corrected values					
Time (s)	Mean W	C (mL×kg^− 1^×min^− 1^)	Mean VO_2_ demand (mL×kg^− 1^×min^− 1^)	Mean VO_2_ (mL×kg^− 1^×min^− 1^)	MAOD (mL×kg^− 1^)
Uncorrected values					
180	500	0.180	90 × 3 min	75 × 3 min	45
Corrected values					
180	500	0.180	(90 × 3 min)	(75 × 3 min) + 5.6	39.4

The 5.6 mL×kg^− 1^ VO_2_ added in the corrected values was based on the 0.25 mMol×kg^− 1^, presented in Hermansen (1974), and used in Medbø and Tabata (1989). Recalculated, the 0.25 mMol×kg^− 1^ accounts for 5.6 mL×kg^− 1^ VO_2_.

MAOD was expressed both as the product of this difference and time, mL×kg^− 1^ and the difference per minute, expressed as mL×kg^− 1^×min^− 1^, as well as relative to VO_2peak_ (% VO_2peak_). To calculate VO_2_ demand, the product of mean TT power and the oxygen cost, i.e., W × C was used (Billat et al. 2009).

### Statistics

Normal distribution was found for 10 s (*p* = 0.25), 30 s (*p* = 0.42), 60 s (*p* = 0.18), 120 s (*p* = 0.60) and 180 s (*p* = 0.13) by using Shapiro-Wilk test and QQ-plots. The descriptive data (Table 1) are therefore presented as mean ± standard deviation (SD) and coefficient of variance (CV %). Statistical power calculations to determine the sample size were partly based on the expected difference in measures of MAOD between TT60 and TT120. Based on measurements from Medbø and Tabata (1989), we expected that the largest increase in VO_2_ and the largest decrease in MAOD per minute would occur between 60 and 120 s. We also conducted power calculations regarding correlations between power output during 180TT and MAP. Power was set to 80% with a significance level of 0.05. From these different calculations we would need a total of 15 and 17 participants, respectively.

The slowest and fastest cyclists were divided by being above or below mean W in 180TT and included all cyclists together. Differences between the slowest and fastest cyclists were analyzed by an independent sample t-test, while paired sample t-test was used to compare means of different variables in all time trials.

Linear regression analyses were used to evaluate possible relationships between the 180 s or MAOD and the other variables. A p value < 0.05 was accepted as statistically significant in all tests. All correlations were expressed as the correlation factor r from Pearson’s bivariate tests (correlation tests) and supplemented with the standard error of the estimate (SEE), as well as 95% CI in tables and – or text. Correlation r-values were categorized as, high, 0.8–0.9; moderate, 0.7–0.8; low, 0.6–0.7 (Stöggl et al. 2007). Multiple and stepwise regressions were considered but were not included mainly for two reasons. The material is underpowered in order to perform multiple regression. Several of the combinations of variables would end up with only two or three participants included. Additionally, when performing multiple and stepwise regression, as expected, co-linearity appeared between several of the variables, e.g., between MAOD and MSP with a variation inflation factor (VIF) as high as 20. Statistical calculations and analyses were performed using the software program STATA version 18.0 BE (Statistics and Data Science, StataCorp, College Station, Texas, USA), while figures were made with GraphPad Prism version 10.4.1.

## Results

**Fig. 1:**
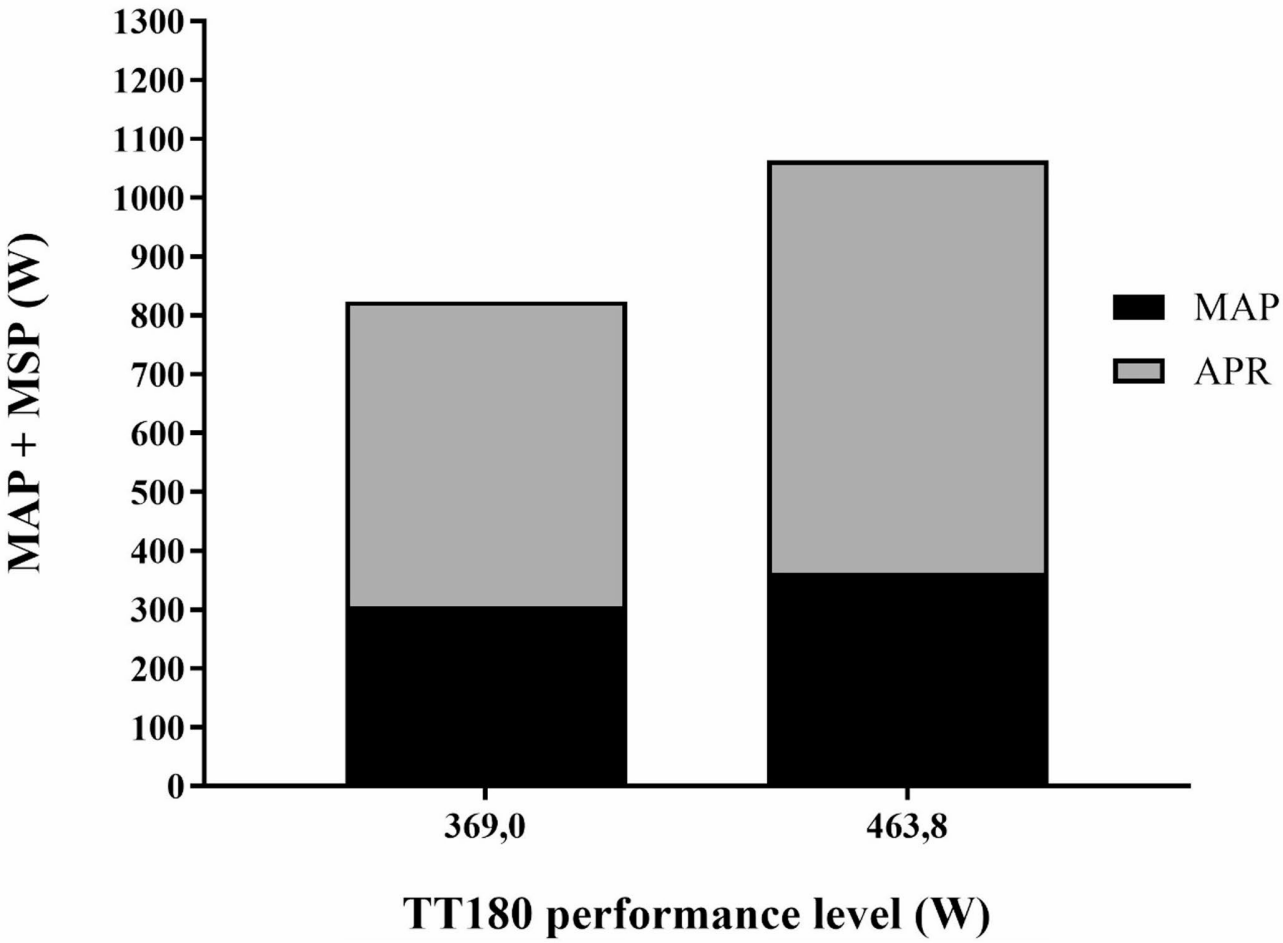
Differences in performance level in TT180. The figure shows 180TT performance level in W on the X-axis, and MAP + MSP in W on the Y-axis. The bars show two groups, lowest performing group (369 W) and highest performing group (463.8), divided based on mean W performance in 180TT. APR is the difference between MAP and MSP

**Fig. 2:**
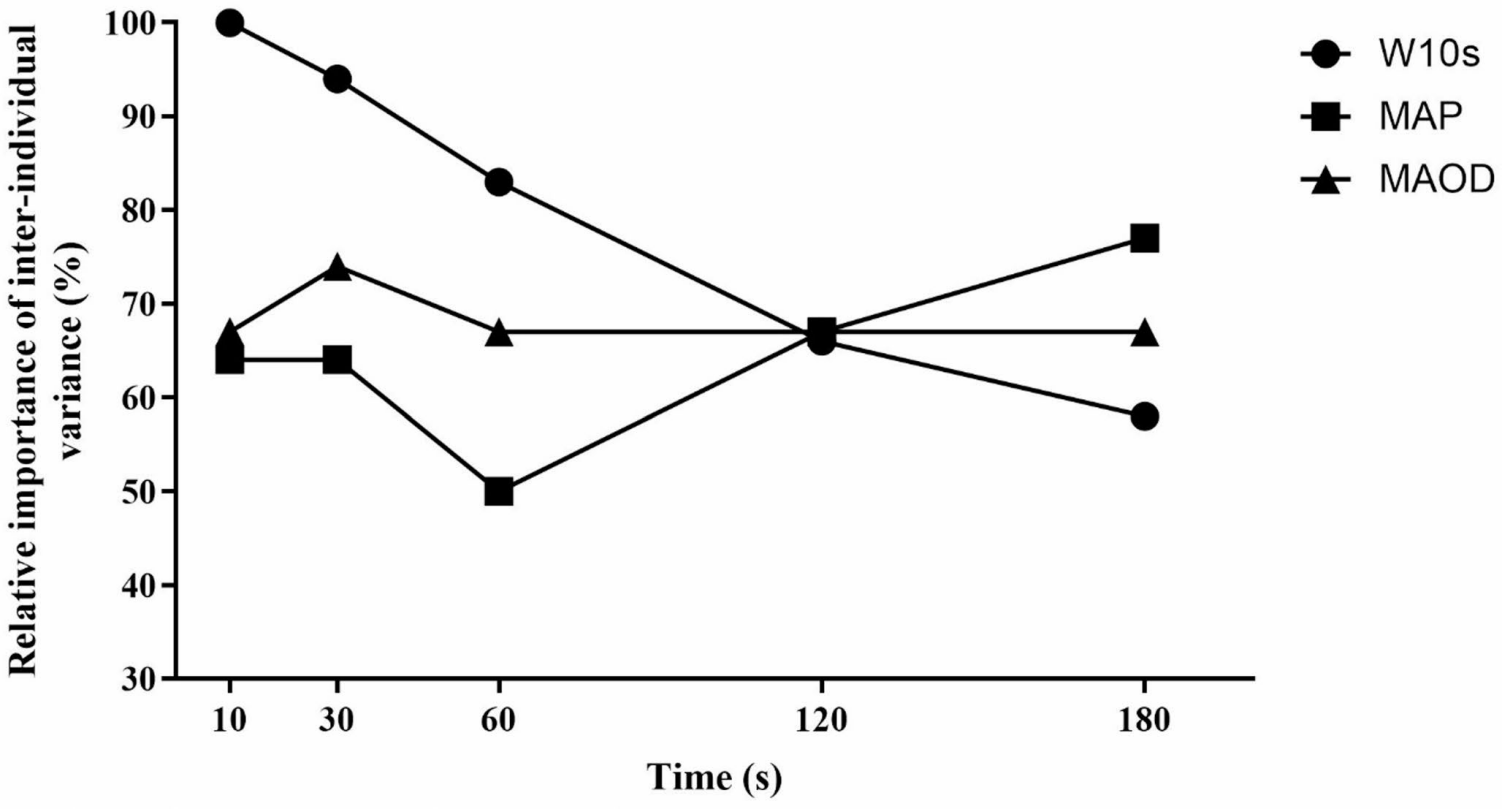
Relative importance of inter-individual variance. The figure shows the relative importance of inter-individual variance in W10 s (MSP), MAP and MAOD in the different time trials, with relative importance of inter-individual variance in % on the X-axis and time in seconds on the Y-axis

Subject characteristics and results are presented in Table 1. Performance and physiological results at baseline are presented in Table 2, divided into two groups based on 180TT mean power performance. There was a significant difference between the cyclists with the lowest (< 422 W) and highest (> 422 W) values in W performance (Fig. 1) and in MAP, MSP, APR and MAODcorr at 180TT (*p* < 0.01), but no significant difference in C and relative energy distribution. The relative importance of inter-individual variance in 180TT was shown in Fig. 2.

**Table 2:** Performance and physiological results at baseline (*N*=18)

	180TT < 422 W (*N*=8)		180TT > 422 W (*N*=10)
VO_2peak_			
L⋅min^-1^	4.50 ± 0.51 (11.3)		5.27 ± 0.30 (5.7)**
mL⋅kg^-1^⋅min^-1^	53.8 ± 10.0 (18.7)		62.5 ± 5.2 (8.3)*
C			
mL⋅kg^-1^⋅W^-1^	0.176 ± 0.029 (16.3)		0.172 ± 0.011 (6.7)
MAP			
W	306.1 ± 30.0 (9.8)		362.7 ± 16.0 (4.4)**
MSP			
W	824.2 ± 112.5 (13.6)		1063.3 ± 78.3 (7.4)**
APR			
W	518.1 ± 111.1 (21.4)		700.6 ± 68.3 (9.8)**
0.7MAP + 0.3MSP W	461.5 ± 42.8 (9.3)		572.9 ± 32.2 (5.6)**
MAODcorr 180TT			
mL⋅kg^-1^	53.0 ± 9.9 (18.8)		73.6 ± 12.6 (17.1)**
mL⋅kg^-1^⋅min^-1^	13.9 ± 3.3 (23.9)		20.8 ± 4.2 (20.2)**
%VO_2peak_	99.8 ± 15.3 (15.4)		118.3 ± 21.0 (17.7)
[La^-^]_b_ 180TT	13.1 ± 2.6 (19.9)		15.6 ± 1.9 (12.3)*
Energy distribution 180TT			
Aerobic metabolism (%) Anaerobic metabolism (%)	73 ± 2.9 (4.1) 27 ± 2.9 (10.7)		69 ± 3.9 (5.7) 31 ± 3.9 (12.9)

Values are mean ± standard deviation, with coefficient of variance in per cent in parenthesis. kg, kilogram. VO_2peak_, peak oxygen consumption. C, oxygen cost of cycling. W, watts. MAP, maximal aerobic power (VO_2peak_ / C). MSP, maximal sprint power. APR, anaerobic power reserve. [La^-^]_b_, blood lactate concentration in millimole⋅L^-1^ (mM). MAOD, maximal accumulated oxygen deficiency. MAODcorr, corrected MAOD

*p < 0.05 different from > 422 W

**p < 0.01 different from > 422 W

**Fig. 3:**
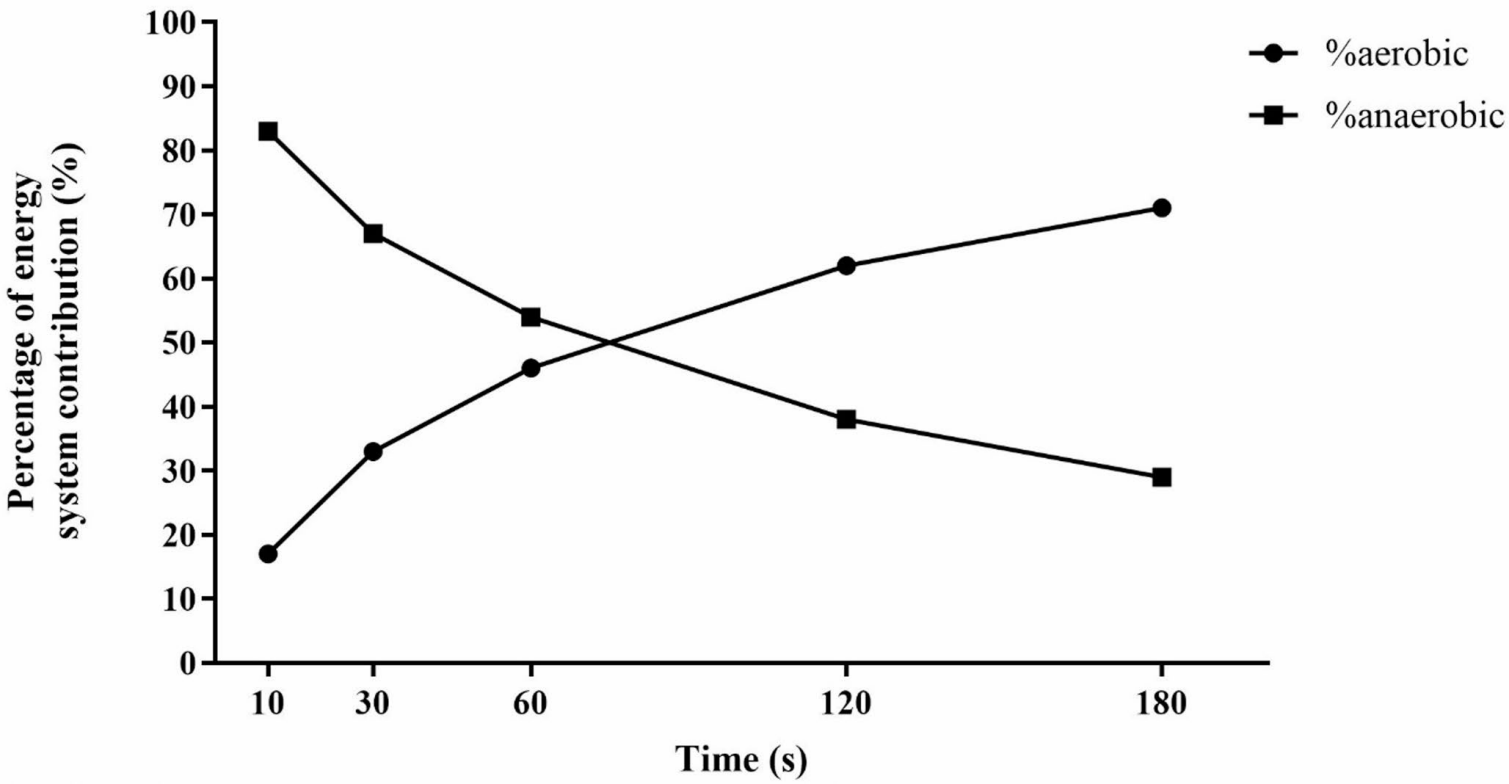
Percentage energy system contribution. The figure shows mean percentage energy contribution on the different time trials, with percentage of system contribution in % from aerobic and anaerobic energy systems on the X-axis and time in seconds on the Y-axis

The relative contribution of aerobic and anaerobic work in the different time trials is presented in Table 3 and shown in Fig. 3. A 50–50% distribution of anaerobic and aerobic energy release was found at 73 s.

**Table 3:** Relative (%) distribution of aerobic and anaerobic work (*N*=18)

	MAODcorr (mL·kg^-1^)	VO_2_ demand (mL·kg^-1^)	%aerobic	%anaerobic
10 s	22.8 ± 4.0 (17.6)	27.5 ± 4.2 (15.2)	17	83
30 s	46.2 ± 10.3 (22.3)	68.6 ± 10.0 (14.6)	33	67
60 s	57.0 ± 10.3 (18.1)	106.0 ± 13.3 (12.5)	46	54
120 s	62.8 ± 12.7 (20.3)	163.8 ± 23.1 (14.1)	62	38
180 s	64.5 ± 15.4 (23.8)	219.1 ± 33.3 (15.2)	71	29

Values are mean ± standard deviation, with coefficient of variance in per cent in parenthesis

Baseline correlations with single variables and performance in 180TT are presented in Table 4. When performance was expressed in absolute watt values, strong positive correlations were found between performance in 180TT and MSP (*r* = 0.85, *p* < 0.01, 95% CI [0.55, 0.93]), MAP (*r* = 0.88, *p* < 0.01, 95% CI [0.69, 0.95], MAOD expressed in mL· kg-^1^ (*r* = 0.81, *p* < 0.01, 95% CI [0.56, 0.93]) and 0.7MAP + 0.3MSP (*r* = 0.91, *p* < 0.01, 95% CI [0.77, 0.97]). VO_2peak_ (L·min^− 1^) correlated strongly (*r* = 0.88, *p* < 0.01, 95% CI [0.69, 0.95]) with performance in 180TT, while no correlation was found between C and 180TT performance. A moderate correlation was found between 180TT performance and APR (*r* = 0.77, *p* < 0.01, 95% CI [0.47, 0.91]). Baseline correlations with single variables and 10TT, 30TT, 60TT, and 120TT are presented in Supplementary Table 1.

**Table 4:** Correlations with performance (W) in 180TT (*N*=18)

	W	W·kg^–1^
VO_2peak_		
L·min^-1^	0.88 (27.1) (0.71, 0.96)**	0.61 (0.6) (0.21, 0.84)**
mL·kg^-1^·min^-1^	0.49 (50.1) (0.03, 0.78)*	0.91 (0.3) (0.77, 0.97)**
mL·kg^-0.67^·min^-1^	0.66 (43.4) (0.27, 0.86)**	0.90 (0.3) (0.77, 0.97)**
C		
mL·kg^-1^·W^-1^	-0.19 (56.4) (-0.61, 0.30)	0.51 (0.7) (0.06, 0.79)*
mL·kg^-0.67^·W^-1^	-0.15 (56.8) (-0.58, 0.34)	0.47 (0.7) (0.02, 0.77)*
MAP (W)	0.88 (27.7) (0.69, 0.95)**	0.76 (0.5) (0.46, 0.91)**
MSP (W)	0.85 (30.2) (0.64, 0.94)**	0.46 (0.7) (-0.01, 0.76)
APR		
W	0.77 (36.7) (0.47, 0.91)**	0.34 (0.8) (-0.16, 0.69)
%VO_2peak_	0.34 (54.1) (-0.16, 0.69)	-0.15 (0.8) (-0.58, 0.34)
MAOD (mL·kg^-1^)	0.81 (33.4) (0.56, 0.93)**	0.71 (0.6) (0.37, 0.89)**
0.7MAP + 0.3MSP	0.91 (23.5) (0.78, 0.97)**	0.61 (0.6) (0.19, 0.84)**

Values are the correlation coefficient r, with the standard error of estimate in first parenthesis, and confidence interval in second parenthesis. VO_2peak_, peak oxygen consumption. C, oxygen cost of cycling. W, watts. MAP, maximal aerobic power (VO_2peak_ / C). MSP, maximal sprint power. APR, anaerobic power reserve. [La^-^]_b_, blood lactate concentration in millimole⋅L^-1^ (mM). MAOD, maximal accumulated oxygen deficiency

*p<0.05 significant correlation

** *p*<0.01 significant correlation

When 180TT mean watt performance was expressed in W· kg^− 1^, strong correlations were found with VO_2peak_, expressed in mL·kg^− 1^·min^− 1^ (*r* = 0.91, *p* < 0.01, 95% CI [0.77, 0.97]) mL·kg^− 0.67^·min^− 1^ (*r* = 0.90, *p* < 0.01, 95% CI [0.77, 0.97]). Moderate correlations were found with MAP (*r* = 0.76, *p* < 0.01, 95% CI [0.46, 0.91]) and MAOD (*r* = 0.71, *p* < 0.01, 95% CI [0.37, 0.89]).

Table 5 shows single variable correlations with MAOD. Significant correlations (*p* < 0.01) were found in MAOD measurements for all the different time trials. MAOD measurements also correlated significantly (*p* < 0.01) with mean watt performance in all time trials. APR did not correlate with MAOD measurements in 120TT and 180TT (Table 6).

**Table 5:** Correlations with MAOD (mL·kg^-1^) in 10TT and 30TT (*N*=18)

		MAOD 10TT		MAOD 30TT	
MAOD (mL·kg^-1^) 10TT				0.93 (0.82, 0.97)**	
MAOD (mL·kg^-1^) 30TT		0.93 (0.82. 0.97)**			
MAOD (mL·kg^-1^) 60TT		0.86 (0.63, 0.94)**		0.90 (0.76, 0.96)**	
MAOD (mL·kg^-1^) 120TT		0.59 (0.15, 0.82)**		0.64 (0.25, 0.85)**	
MAOD (mL·kg^-1^) 180TT		0.65 (0.27, 0.86)**		0.63 (0.24, 0.85)**	
W_av_ 10TT		0.82 (0.55, 0.93)**		0.87 (0.67, 0.95)**	
W_av_ 30TT		0.78 (0.48, 0.91)**		0.86 (0.67, 0.95)**	
W_av_ 60TT		0.74 (0.39, 0.89)**		0.83 (0.59, 0.93)**	
W_av_ 120TT		0.72 (0.34, 0.88)**		0.80 (0.54, 0.92)**	
W_av_ 180TT		0.80 (0.50, 0.92)**		0.82 (0.58, 0.93)**	
[La^-^]_b_ 60TT		0.70 (0.34, 0.88)**		0.60 (0.19, 0.84)**	
MAP (W)		0.80 (0.49, 0.91)**		0.78 (0.48, 0.91)**	
APR (W)		0.75 (0.43, 0.90)**		0.82 (0.57, 0.93)**	

Values are the correlation coefficient r, with coefficient intervals in parenthesis. W_av_, average watts produced during time trials. MAP, maximal aerobic power (VO_2peak_ / C). APR, anaerobic power reserve. [La^-^]_b_, blood lactate concentration in millimole⋅L^-1^ (mM). MAOD, maximal accumulated oxygen deficiency

*p<0.05 significant correlation

** *p*<0.01 significant correlation

**Table 6:** Correlations with MAOD (mL·kg^-1^) in 60TT, 120TT and 180TT (*N*=18)

	MAOD 60TT	MAOD 120TT	MAOD 180TT
MAOD (mL·kg^-1^) 10TT	0.86 (0.63, 0.94)**	0.59 (0.15, 0.82)**	0.65 (0.27, 0.86)**
MAOD (mL·kg^-1^) 30TT	0.90 (0.76, 0.96)**	0.64 (0.25, 0.85)**	0.63 (0.24, 0.85)**
MAOD (mL·kg^-1^) 60TT		0.76 (0.46, 0.91)**	0.62 (0.22, 0.85)**
MAOD (mL·kg^-1^) 120TT	0.76 (0.46, 0.91)**		0.76 (0.46, 0.91)**
MAOD (mL·kg^-1^) 180TT	0.62 (0.22, 0.85)**	0.76 (0.46, 0.91)**	
W_av_ 10TT	0.75 (0.44, 0.90)**	0.53 (0.08, 0.80)*	0.52 (0.06, 0.79)*
W_av_ 30TT	0.76 (0.46, 0.91)**	0.51 (0.06, 0.79)*	0.49 (0.03, 0.78)*
W_av_ 60TT	0.82 (0.58, 0.93)**	0.61 (0.21, 0.84)**	0.52 (0.07, 0.79)*
W_av_ 120TT	0.80 (0.53, 0.92)**	0.81 (0.55, 0.93)**	0.67 (0.30, 0.87)**
W_av_ 180TT	0.78 (0.50, 0.92)**	0.77 (0.47, 0.91)**	0.81 (0.56, 0.93)**
[La^-^]_b_ 60TT	0.69 (0.34, 0.88)**	0.47 (0.00, 0.77)*	0.31 (-0.18, 0.68)
MAP (W)	0.65 (0.26, 0.86)**	0.62 (0.22, 0.84)**	0.62 (0.22, 0.85)**
APR (W)	0.72 (0.38, 0.89)**	0.45 (-0.01, 0.76)	0.44 (-0.04, 0.75)

Values are the correlation coefficient r, with the standard error of estimate in parenthesis. W_av_, average watts produced during time trials. MAP, maximal aerobic power (VO_2peak_ / C). APR, anaerobic power reserve. [La^-^]_b_, blood lactate concentration in millimole⋅L^-1^ (mM). MAOD, maximal accumulated oxygen deficiency

*p<0.05 significant correlation

** *p*<0.01 significant correlation

## Discussion

The main findings in this study were that, on average, there was a 50–50% distribution of aerobic vs. anaerobic energy release at 73s, while at 180 s the distribution was 71 − 29% aerobic vs. anaerobic, respectively. Performance in 180TT was strongly related to MAP, MSP, MAOD and the 0.7MAP + 0.3MSP equation, and with a moderate positive correlation with APR. Since C did not correlate with 180TT, the relationship between MAP and 180TT could primarily be explained by the strong correlation between VO_2peak_ (L·min^− 1^) and 180TT. The findings in this study showed that the best performing cyclists in 180TT had significantly higher values in several of the tested variables, including MAP, MSP, APR and MAOD. Given the strong correlations between both MAP and MSP with 180TT found in this study, it was reasonable that the 0.7MAP + 0.3MSP equation explained approximately 83% of the performance, based on the R^2^-value.

The calculation of the relative contribution of aerobic and anaerobic energy release during all time trials (Table 3) was conducted in this study based on the total VO_2_ demand (mL·kg^− 1^) and MAODcorr (mL·kg^− 1^). Gastin (2001) pointed out that the choice of method may influence how the distribution of aerobic and anaerobic energy contribution is reported. The method used in the present study is the same as was used in Medbø and Tabata (1989). We cannot rule out that any other choice of methods could have resulted in small differences in results. Results from this study regarding the distribution from aerobic and anaerobic energy systems during TTs aligned with findings in Medbø and Tabata (1989), showing that the contribution from aerobic and anaerobic energy production are equal after approximately 70 s of work with maximal effort. The overview of the distribution in both the shorter and longer TTs is largely consistent with the findings of previous studies (Craig and Norton 2001; Spencer and Gastin 2001; Losnegard et al. 2012; Andersson et al. 2017).

For the sample investigated in this study, MAP (*r* = 0.88, *p* < 0.01, 95% CI [0.69, 0.95]) was strongly related to performance in 180TT. The relative contribution to energy production of 71% aerobic work at 180 s, found in this study, highlights the importance of aerobic endurance already in competitions as short as 3 min. In cycling, this would typically be important in track cycling races. The strong relationship between VO_2peak_ and 180TT found in this study aligns with results from other studies examining short cycling time trials (Craig et al. 1993; Babault et al. 2018; Støa et al. 2025). Støa et al. (2025) found significant correlations between performance and both VO_2peak_ and C, while the results in Craig et al. (1993) are similar to the results in the present study, finding performance in a 4000 m time trial to correlate significantly with VO_2peak_ but not with C. The results from the present study are also consistent with other studies finding middle-distance performance to correlate with MAP in running (Ingham et al. 2008; Bellinger et al. 2021^a^; Støren et al. 2021;) and cross-country skiing (Stöggl et al. 2007; Støren et al. 2023; Gjerløw et al. 2025). In more homogenous cohorts in terms of VO_2peak_, Craig and Morgan (1998) and Tanji et al. (2018) did not find VO_2peak_ to correlate with 800 m running. However, Tanji et al. (2018) found a significant correlation between 800 m performance and C in their study.

MSP correlated strongly with 180TT performance (*r* = 0.85, *p* < 0.01, 95% CI [0.64, 0.94]) in this study, and the highest-performing cyclists measured significantly higher MSP (*p* < 0.01) than the lowest-performing cyclists. MSP has in previous studies been related to middle-distance in running (Sandford et al., 2019^a^; Støren et al. 2021; Jimenez-Reyes et al. 2022), cross-country sprint skiing (Stöggl et al. 2007; Støren et al. 2023; Gjerløw et al. 2025) and short time trial cycling (Støa et al. 2025). When MSP is discussed in relation to middle-distance performance, this variable has also been associated with APR (Sandford et al., 2019^b^). Higher APR was related to better performance in 180TT in the present study. del Arco et al. (2022) discussed the impact of APR on performance in middle-distance running and concluded that APR would only relate to performance in cohorts with no or minor differences in MAP or MSP.

Anaerobic endurance capacity, measured as either MAOD or TTE at a supra-maximal intensity related to MAP, as a determinant of performance in middle-distance time trials, has given equivocal findings in existing literature. In the present study, MAOD correlated strongly (*r* = 0.81, *p* < 0.01, 95% CI [0.56, 0.93]) with performance in 180TT. This is consistent with the findings in some studies examining middle-distance performance (Craig et al. 1993; Ramsbottom et al. 1994; Billat et al. 2009; Losnegard et al. 2012) but in contrast with others (Craig and Morgan 1998; Støren et al. 2021, 2023; Støa et al. 2025). When comparing the highest-performing athletes to the lowest-performing athletes, MAOD values were significantly higher for the highest-performing athletes. What was also interesting in the present study was that MAOD measured at 180TT correlated significantly with MAOD measured at all the other time trials, meaning that the cyclists with the highest MAOD at 180TT also had the highest MAOD at all shorter time trials. In Gjerløw et al. (2025), no association was found between MAOD and 800 m ski ergometer TT performance. Furthermore, and in contrast to the results in the present study, there was no significant difference found in MAOD, neither when expressed as mL·kg^− 1^ or mL·kg^− 1^·min^− 1^, when comparing the fastest and the slowest skiers in 800 m ski ergometer TT in Gjerløw et al. (2025).

MAOD expressed as mL·kg^− 1^ was not significantly different between 60TT, 120TT and 180TT in the present study. The results showed significant increases in MAOD from 10TT to 30TT, and from 30TT to 60TT. Even though there was a small, yet not significant, rise in MAOD from TT60 to TT120 and from TT120 to TT180, the curve seems to level off between one and two minutes of work. These findings are not necessarily unexpected, given the results reported by Hill and Vingren (2011), who did not find any difference in MAOD in three all-out cycling and running tests lasting 3, 5 and 7 minutes. Similar results were reported in Spencer and Gastin (2001), who found no further increase in AOD from 800 m to 1500 m running, after measuring increased AOD during 200- 400- and 800 m. The lack of rise in total MAOD after approximately 1–2 minutes, supports the proposal of MAOD being a set limit of anaerobic energy available, presented in Støren et al. (2023). In that study, a strong correlation was found between APR (%MAP) and MAOD (%VO_2peak_), suggesting that MAOD would be the product of an athlete’s APR. Both APR (r = 0.77, p < 0.01, 95% CI [0.47, 0.91]) and MSP (r = 0.85, p < 0.01, 95% CI [0.64, 0.94]) correlated significantly with 180TT in the present study. Our observation that MAOD rises steeply from 10–60 s but shows diminishing gains from 120–180 s is consistent with the critical-power (CP) framework, in which a finite work capacity above CP (W′) is expended rapidly early on and then approaches depletion as task duration lengthens (Bergstrøm et al. 2014). However, MAOD- and CP-based quantities are related but not interchangeable, since MAOD is a volume and W is power. The convergence of our MAOD pattern with the CP/W’ framework supports the interpretation of a limited anaerobic store over short–middle severe-intensity efforts. This further aligns with the model presented in Billat et al. (2009), that the athletes with the highest anaerobic store available are best positioned to portion this anaerobic store out when working at a set supramaximal intensity relative to MAP. Theoretically, when comparing two athletes with similar MAP or CP, the athlete with the higher MSP, and thus the higher APR, should have the greatest potential for anaerobic capacity. Gastin et al. (1994) showed a relationship between peak power output and MAOD.

Correlation analysis only indicates which variables are most strongly associated within the sample on which the analyses are conducted. In the present study, both MAP (*r* = 0.88, *p* < 0.01, 95% CI [0.69, 0.95]) and MAOD (*r* = 0.81, *p* < 0.01, 95% CI [0.56, 0.93]) correlated strongly with performance in 180TT and could therefore be seen as almost equally statistically important for performance. However, this may be misleading, as calculations of the relative contributions from aerobic and anaerobic energy release after 180 s (Table 3; Fig. 1) indicated a 71/29% split, respectively. This implies that MAP should be of relatively greater importance for performance in three minutes of maximal effort than anaerobic capacity (i.e., MAOD).

### Practical implications, limitations, and future perspectives

The combined predictor 0.7MAP + 0.3MSP should be viewed as a data-driven, post-hoc heuristic derived in this specific sample rather than a mechanistic law. With our modest n and multiple correlated predictors, the apparent weighting may reflect sample-specific structure and may not generalize.

The results from the present study suggest focusing on MAP and MSP as short-time trial performance variables in cycling. An increase in MAP can be achieved by improving VO_2peak_ or C. VO_2peak_ in cyclists has been shown to be effectively increased by focusing on high intensity interval training (Sylta et al. 2016; Rønnestad et al. 2021) while C has previously been shown to improve with maximal strength training (Sunde et al. 2010; Vikmoen et al. 2015). To improve MSP, both short-sprint training and/or maximal strength training are shown to be effective (Kristoffersen et al. 2019). However, training prescription must be individualized: the optimal balance between MAP and MSP plausibly varies with athlete phenotype, technique/cadence, fatigue state, and event duration.

We cannot rule out that the relatively short break of 30 min could have affected the results of the TTs in this study. However, pilot testing showed little or no decrease in power output at a given TT 30 min after another test. Only small decreases in power output were found in Almquist et al. (2021), who used 4-minute breaks between 30 s repeated sprints.

Although the results of this study show a statistical association between short time cycling performance and physiological variables such as MAP, MSP, and MAOD, causality cannot be established based on these correlations alone. Future studies could pre-register model structure, validate the weights out-of-sample (e.g., cross-validation or external cohort), and report prediction intervals. Additionally, intervention studies should be conducted to further investigate the cause-response relationship.

## Conclusions

The results from the present study show that in 180 s all-out work, the energy expenditure was 71% dependent on aerobic energy release, and 29% dependent on anaerobic release. MAP, MSP and MAOD were all related to performance in the present sample. Our findings suggest that anaerobic capacity measured as MAOD seems to be highly dependent on the athlete’s MSP. MAOD increased rapidly between 10 and 60 s and showed little additional rise from 120 to 180 s. Interpreted within the critical-power/W′ framework, this pattern supports the existence of a finite work capacity above CP over these severe-intensity efforts. The 0.7MAP + 0.3MSP model showed a strong relationship with watt performance in 180 s, but is a post-hoc heuristic model, and its practical use should remain individualized and subject to external validation. We suggest that training to improve MAP and MSP should be implemented when planning training for enhanced performance.

## Supplementary Information

Below is the link to the electronic supplementary material.

Supplementary file1

## Data Availability

The data sets generated during and/or analyzed during the current study are available from the corresponding author on reasonable request.

## Abbreviations

AOD: Accumulated oxygen deficit
APR: Anaerobic power reserve
ASR: Anaerobic sprint reserve
BM: Body mass
C: Oxygen cost of cycling
HR: Heart rate
HR_peak_: Peak heart rate
[La^−^]_b_: Blood lactate concentration
MAOD: Maximal accumulated oxygen deficit
MAODcorr: Corrected maximal accumulated oxygen deficit
MAP: Maximal aerobic power
MAS: Maximal aerobic speed
MSP: Maximal sprint power
MSS: Maximal sprint speed
RER: Respiratory exchange ratio
TT: Time trial
TTE: Time to exhaustion
VO_2peak_: Peak oxygen uptake
W: Watt

## References

[CR1] Almquist NW, Sandbakk Ø, Rønnestad BR, Noordhof D (2021) The aerobic and anaerobic contribution during repeated 30-s sprints in elite cyclists. Front Physiol 2021(12):692622. 10.3389/fphys.2021.692622 10.3389/fphys.2021.692622 PMC8187900 34122152

[CR2] Andersson E, Bjorklund G, Holmberg HC, Ortenblad N (2017) Energy system contributions and determinants of performance in sprint cross-country skiing. Scand J Med Sci Sports 27:385–398. 10.1111/sms.12666 26923666 10.1111/sms.12666

[CR3] Babault N, Poisson M, Cimadoro G, Cometti C, Païzis C (2018) Performance determinants of fixed gear cycling during criteriums. Eur J Sport Sci 18(9):1199–1207. 10.1080/17461391.2018.1484177 29911491 10.1080/17461391.2018.1484177

[CR4] Bellinger P, Derave W, Lievens E, Kennedy B, Arnold B, Rice H, Minahan C (2021) Determinants of performance in paced and maximal 800-m running time trials. Med Sci Sports Exerc 12:2635–2644. 10.1249/mss.0000000000002755 10.1249/MSS.0000000000002755 34310491

[CR5] Bentley DJ, Wilson GJ, Davie AJ et al (1998) Correlation between peak power output, muscular strength and cycle time trial performance in triathletes. *J Sports Med Phys Fitness.* 1998;38:201-7 9830826

[CR6] Bergstrom HC, Housh TJ, Zuniga JM, Traylor DA, Lewis RW, Camic CL, Schmidt RJ, Johnson GO (2014) Differences among estimates of critical power and anaerobic work capacity derived from five mathematical models and the three-minute all-out test. J Strength Cond Res 28:592–600 24566607 10.1519/JSC.0b013e31829b576d

[CR7] Billat V, Hamard L, Koralsztein JP, Morton RH (2009) Differential modeling of anaerobic and aerobic metabolism in the 800-m and 1,500-m run. J Appl Physiol (1985) 107(2):478–487. 10.1152/japplphysiol.91296.2008 19478190 10.1152/japplphysiol.91296.2008

[CR43] Blondel N, Berthoin S, Billat V, Lensel G (2001) Relationship Between Run Times to Exhaustion at 90, 100, 120, and 140% of vV˙O2max and Velocity Expressed Relatively to Critical Velocity and Maximal Velocity. Int J Sports Med 22(1):27–33. https://doi.org/10.1055/s-2001-11357 10.1055/s-2001-11357 11258638

[CR8] Craig IS, Morgan DW (1998) Relationship between 800-m running performance and accumulated oxygen deficit in middle-distance runners. Med Sci Sports Exerc 30:1631–1636. 10.1097/00005768-199811000-00012 9813877 10.1097/00005768-199811000-00012

[CR9] Craig NP, Norton KI (2001) Characteristics of Track Cycling. Sports Med. 2001;31:457–468. 10.2165/00007256-200131070-00001 10.2165/00007256-200131070-00001 11428683

[CR10] Craig NP, Norton KI, Bourdon PC, Woolford SM, Stanef T, Squires B et al (1993) Aerobic and anaerobic indices contributing to track endurance cycling performance. Eur J Appl Physiol Occup Physiol 67(2):150–158. 10.1007/BF00376659 8223521 10.1007/BF00376659

[CR11] del Arco A, Aguirre-Betolaza AM, Castaneda-Babarro A (2022) Anaerobic speed reserve and middle-distance performance: a systematic review. Strength Cond J. https://doi.org/10.1519/SSC.00000

[CR12] Driss T, Vandewalle H (2013) The measurement of maximal (anaerobic) power output on a cycle ergometer: a critical review. Biomed Res Int 2013:589361. 10.1155/2013/58936 24073413 10.1155/2013/589361 PMC3773392

[CR42] Erik W, Faria Daryl L, Parker Irvin E, Faria (2005) The Science of Cycling Sports Medicine 35(4):285–312. https://doi.org/10.2165/00007256-200535040-00002 10.2165/00007256-200535040-00002 15831059

[CR13] Gastin PB (2001) Energy system interaction and relative contribution during maximal exercise. Sports Med 31:725–741 11547894 10.2165/00007256-200131100-00003

[CR14] Gjerløw LE, Sunde A, Støa EM, Helgerud J, Johansen J-M, Hjortland H, Støren Ø (2025) Determining physiologic variables for changes in 800 m running and 800 m ski ergometer performance. Eur J Appl Physiol. 10.1007/s00421-025-05765-7 40251330 10.1007/s00421-025-05765-7 PMC12479658

[CR15] Hallam LC, Ducharme JB, Mang ZA, Amorim FT (2022) The role of the anaerobic speed reserve in female middle-distance running. Sci Sport 37: 637–675,2022

[CR16] Hermansen L (1974) Oxygen transport during exercise in human subjects. Acta Physiol Stand 90:l–104 4522516

[CR17] Hill DW, Vingren JL (2011) Maximal accumulated oxygen deficit in running and cycling. Appl Physiol Nutr Metab 36:831–838. 10.1139/h11-108 22050108 10.1139/h11-108

[CR18] Ingham SA, Whyte GP, Pedlar C, Bailey DM, Dunman N, Nevill AM (2008) Determinants of of 800-m and 1500-m running performance using allometric models. Med Sci Sports Exerc 40:345–350. 10.1249/mss.0b013e31815a83dc 18202566 10.1249/mss.0b013e31815a83dc

[CR19] Jimenez-Reyes P, Cuadrado-Penafiel V, Parraga-Montilla JA, Romero-Franco N, Casado A (2022) Anaerobic speed reserve, sprint force-velocity profile, kinematic characteristics, and jump ability among elite male speed- and endurance-adapted milers. *Int J Environ Res Public Health* 19: 1447–11458, 2022 10.3390/ijerph19031447 PMC8835231 35162462

[CR20] Kristoffersen M, Sandbakk Ø, Rønnestad BR, Gundersen H (2019) Comparison of short-sprint and heavy strength training on cycling performance. Front Physiol 10:1132. 10.3389/fphys.2019.01132 31555153 10.3389/fphys.2019.01132 PMC6724228

[CR21] Losnegard T, Hallén J (2014) Physiological differences between sprint- and distance specialized cross-country skiers. Int J Sports Physiol Perform 9(1):25–31. 10.1123/ijspp.2013-0066 24155024 10.1123/ijspp.2013-0066

[CR22] Losnegard T, Myklebust H, Hallén J (2012) Anaerobic capacity as a determinant of performance in sprint skiing. Med Sci Sports Exerc 44(4):673–681. 10.1249/MSS0b013 e3182 388684 21952633 10.1249/MSS.0b013e3182388684

[CR23] Medbø JI, Tabata I (1989) Relative importance of aerobic and anaerobic energy release during short-lasting exhausting bicycle exercise. J Appl Physiol 67:1881–1886. 10.1152/jappl.1988.64.1.50 2600022 10.1152/jappl.1989.67.5.1881

[CR24] Noordhof DA, de Koning JJ, Foster C (2010) The maximal accumulated oxygen deficit method: a valid and reliable measure of anaerobic capacity? Sports Med. 2010;40(4):285–302. PubMed 10.2165/11530390-000000000-00000 10.2165/11530390-000000000-00000 20364874

[CR25] Ramsbottom R, Nevill AM, Nevill ME, Newport S, Williams C (1994) Accumulated oxygen deficit and short-distance running performance. J Sports Sci 12(5):447–453. https://doi.org/10.1080/0264041940 7799473 10.1080/02640419408732194

[CR26] Rønnestad BR, Øfsteng SJ, Zambolin F, Raastad T, Hammarström D (2021) Superior physiological adaptations after a microcycle of short intervals versus long intervals in cyclists. Int J Sports Physiol Perform 16(10):1432–1438. 10.1123/ijspp.2020-0647 33735833 10.1123/ijspp.2020-0647

[CR29] Sandford GN, Stellingwerff T (2019) Question your categories: the misunderstood complexity of middle-distance running profiles with implications for research methods and application. FrontSport Act Living 2019(1):1–8 10.3389/fspor.2019.00028 PMC7739647 33344952

[CR27] Sandford GN, Allen SV, Kilding AE, Ross A, Laursen PB (2019a) ^a^ Anaerobic speed reserve: a key component of elite male 800-m running. *Int J Sports Physiol Perform*. 2019;14:501–8 10.1123/ijspp.2018-0163 30300023

[CR28] Sandford G, Kilding A, Ross A, Laursen P (2019b) )^b^. Maximal sprint speed and the anaerobic speed reserve domain; the untapped tools that differentiate the world’s best 800m runners. Sport Med 2019(49):843–852 10.1007/s40279-018-1010-5 30374943

[CR44] Saunders PU, Pyne DB, Telford RD, Hawley JA (2004) Reliability and variability of running economy in elite distance runners. Med Sci Sports Exerc 36(11):1972–6. https://doi.org/10.1249/01.mss.0000145468.17329.9f 15514515 10.1249/01.mss.0000145468.17329.9f

[CR30] Spencer MR, Gastin PB (2001) Energy system contribution during 200- to 1500-m running in highly trained athletes. Med Sci Sports Exerc. 2001;33:157–62 10.1097/00005768-200101000-00024 11194103

[CR31] Støa EM, Rønnestad B, Helgerud J, Johansen J-M, Andersen IT, Rogneflåten T, Sørensen A, Støren Ø (2025) Short-time cycling performance in young elite cyclists: related to maximal aerobic power and not to maximal accumulated oxygen deficit. Front Physiol 15:1536874. 10.3389/fphys.2024.1536874 39867226 10.3389/fphys.2024.1536874 PMC11757254

[CR32] Stöggl T, Lindinger S, Müller E (2007) Analysis of a simulated sprint competition in classical cross country skiing. Scand J Med Sci Sports 17:362–372. 10.1111/j.1600-0838.2006.00589.x 16911588 10.1111/j.1600-0838.2006.00589.x

[CR33] Stöggl R, Müller E, Stöggl T (2015) Motor abilities and anthropometrics in youth cross-country skiing. Scand J Med Sci Sports 25(1):e70–e81. 10.1111/sms.12254 24894129 10.1111/sms.12254

[CR36] Støren Ø, Ulevåg K, Larsen MH, Støa eM, Helgerud J (2013) Physiological determinants of the cycling time trial. J Strength Cond Res 27(9):2366–2373. 10.1519/JSC.0b013e31827f5427 23238091 10.1519/JSC.0b013e31827f5427

[CR34] Støren Ø, Helgerud J, Johansen JM, Gjerløw LE, Aamlid A, Støa EM (2021) Aerobic and anaerobic speed predicts 800 meter running performance in recreational runners. Front Physiol 12:672141. 10.3389/fphys.2021.672141 34093233 10.3389/fphys.2021.672141 PMC8176219

[CR35] Støren Ø, Sunde A, Helgerud J, Johansen JM, Gjerløw LE, Hjortland H, Støa EM (2023) Maximal aerobic and anaerobic power and time performance in 800 m double poling ergometer. Eur J Appl Physiol. 10.1007/s00421-023-05149-9 36750479 10.1007/s00421-023-05149-9 PMC10192160

[CR37] Sunde A, Støren Ø, Bjerkaas M, Larsen MH, Hoff J, Helgerud J (2010) Maximal strength training improves cycling economy in competitive cyclists. J Strength Cond Res 24(8):2157–2165. 10.1519/JSC.0b013e3181aeb16a 19855311 10.1519/JSC.0b013e3181aeb16a

[CR38] Sylta Ø, Tønnessen E, Hammarstrom D, Danielsen J, Skovereng K, Ravn T et al (2016) The effect of different high-intensity periodization models on endurance adaptations. Med Sci Sports Exerc 48:2165–2174 27300278 10.1249/MSS.0000000000001007

[CR39] Tanji F, Tsuji T, Shimazu W, Nabekura Y (2018) Relationship between 800-m running performance and aerobic and anaerobic energy metabolism capacities in well-trained middle- distance runners. Int J Sport Health Sci 16:70–76. 10.5432/ijshs.201724

[CR40] Vikmoen O, Ellefsen S, Trøen Ø, Hollan I, Hanestadhaugen M, Raastad T, Rønnestad BR (2015) Strength training improves cycling performance, fractional utilization of VO2max and cycling economy in female cyclists. Scand J Med Sci Sports 26:384–396 25892654 10.1111/sms.12468

[CR41] Watanabe T, Inaba T, van Rassel CR, MacInnis MJ, Kakinoki K, Hatta H (2024) Identifying physiological determinants of 800 m running performance using post-exercise blood lactate kinetics. Eur J Appl Physiol. 10.1007/s00421-024-05504-4 38761193 10.1007/s00421-024-05504-4 PMC11467099

