# Supplementary material for: Physiological determinants and energy system contribution in short- and middle-distance cycling performance
Source: Eur J Appl Physiol. 2025 Dec 19;126(5):2501–13. doi: 10.1007/s00421-025-06049-w (PMC13236797; doi:10.1007/s00421-025-06049-w)
Supplement: Supplementary file 1 — Supplementary file1 [file 421_2025_6049_MOESM1_ESM.docx]

**Supplementary material**


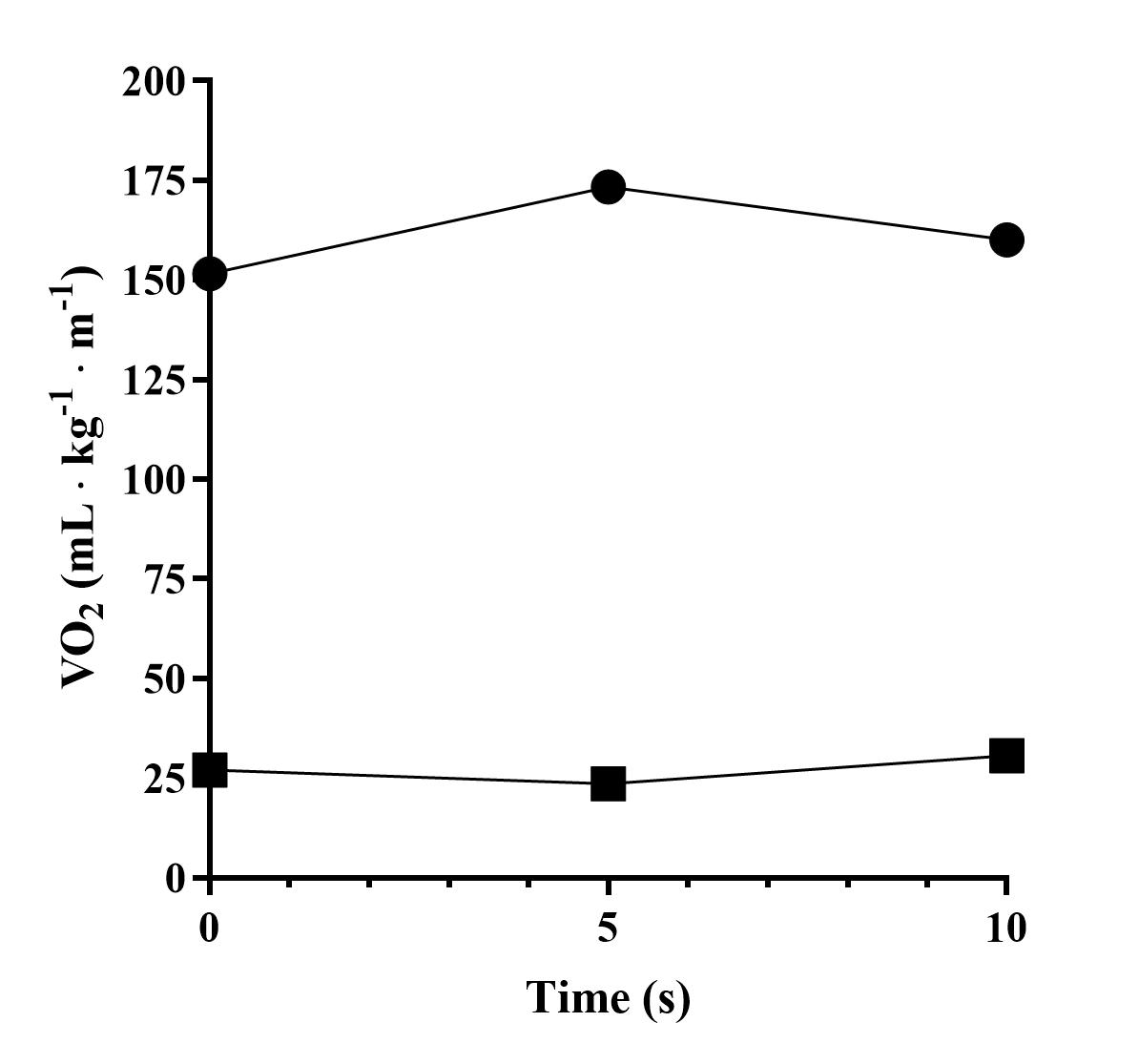


**Supplementary figure 1. Oxygen recordings in 10TT.**

The figure shows one participant during the 10 s recording. It shows time in seconds on the X-axis, and VO_2_ (mL·kg^-1^·min^-1^) on the Y-axis. The upper line shows VO_2_ demand, and the lower line shows VO_2_ measured. VO_2_ demand is the product of oxygen cost of cycling (C) and W at each time point. The area between the two lines denotes the MAOD.


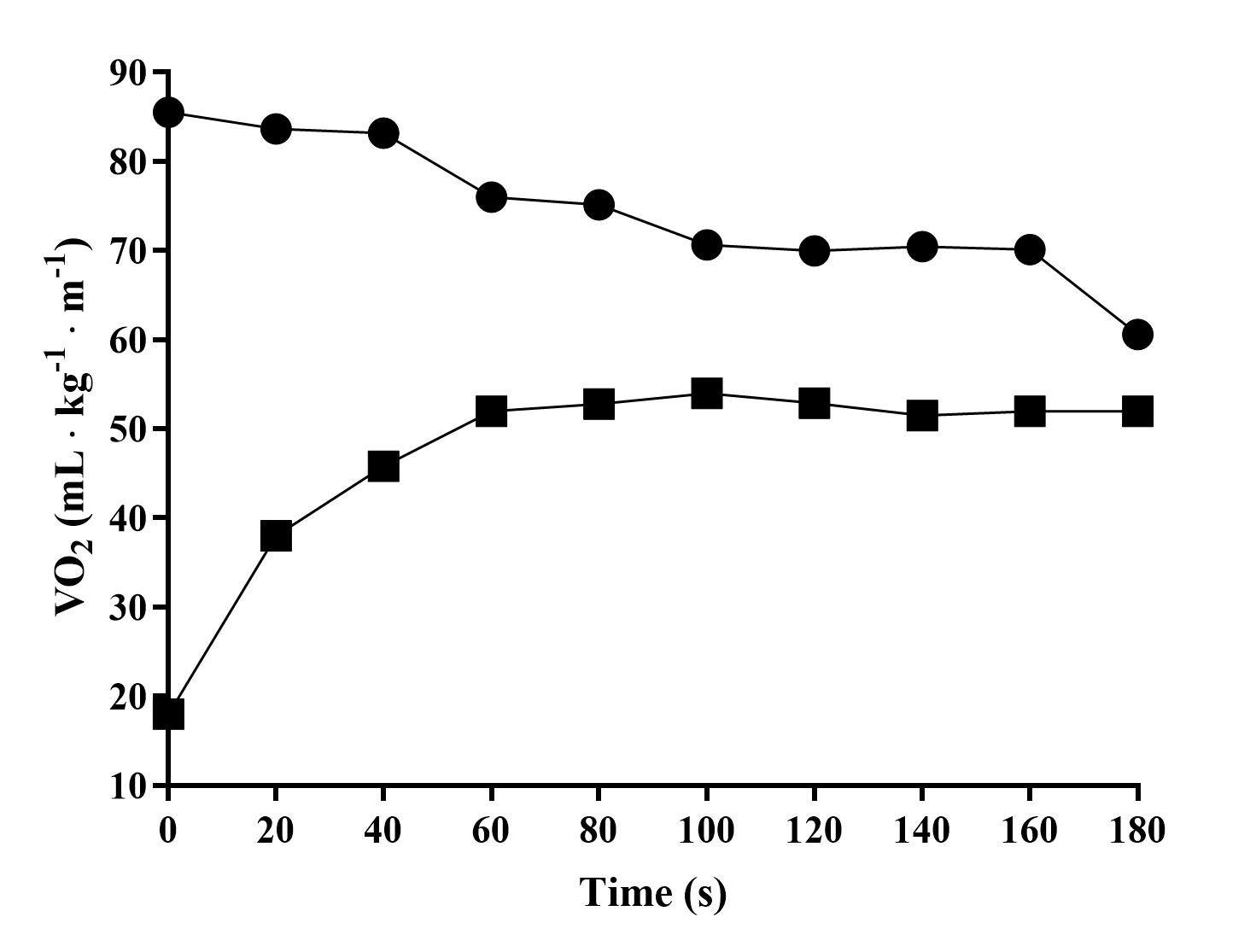


**Supplementary Figure 2. Oxygen recordings in 180TT.**

Figure shows one participant during the 180 s recording. It shows time in seconds on the X-axis, and VO_2_ (mL·kg^-1^·min^-1^) on the Y-axis. The upper line shows VO_2_ demand, and the lower line shows VO_2_ measured. VO_2_ demand is the product of oxygen cost of cycling (C) and W at each time point. The area between the two lines denotes the MAOD.

| **Supplementary Table 1**. **Correlations with performance (W) in 10TT, 30TT, 60TT, 120TT and 180TT** | | | | | |
| --- | --- | --- | --- | --- | --- |
|  | 10TT | 30TT | 60TT | 120TT | 180TT |
| **VO2peak** |  |  |  |  |  |
| L·min^-1^ | 0.67**  (0.28, 0.86) | 0.71**  (0.34, 0.88) | 0.74**  (0.39, 0.89) | 0.87**  (0.66, 0.95) | 0.88**  (0.69, 0.95) |
| mL·kg^-1^·min^-1^ | 0.22  (-0.29, 0.62) | 0.20  (-0.30, 0.60) | 0.16  (-0.34, 0.58) | 0.39  (-0.10, 0.72) | 0.49*  (0.01, 0.77) |
| mL·kg^-0.67^·min^-1^ | 0.38  (-0.12, 0.71) | 0.37  (-0.13, 0.71) | 0.35  (-0.15, 0.70) | 0.57*  (0.13, 0.82) | 0.66**  (0.25, 0.85) |
| **C** |  |  |  |  |  |
| mL·kg^-1^·W^-1^ | -0.47  (-0.76, 0.13) | -0.49*  (-0.78, -0.02) | -0.51*  (-0.78, -0.04) | -0.28  (-0.65, 0.23) | -0.19  (-0.60, 0.31) |
| mL·kg^-0.67^·W^-1^ | -0.48*  (-0.77, -0.01) | -0.49*  (-0.77, -0.01) | -0.47*  (-0.76, 0.01) | -0.22  (-0.62, 0.28) | -0.15  (-0.58, 0.34) |
| **MAP** | 0.76**  (0.45, 0.90) | 0.76**  (0.45, 0.90) | 0.71**  (0.36, 0.88) | 0.82**  (0.58, 0.93) | 0.88**  (0.69, 0.95) |
| **MSP** | 1** | 0.97**  (0.92, 0.99) | 0.91**  (0.78, 0.97) | 0.82**  (0.56, 0.93) | 0.85**  (0.55, 0.93) |
| **APR** |  |  |  |  |  |
| W | 0.98**  (0.95, 0.99) | 0.95**  (0.86, 0.98) | 0.89**  (0.72, 0.96) | 0.74**  (0.42, 0.90) | 0.77**  (0.47, 0.91) |
| %VO_2peak_ | 0.70**  (0.33, 0.87) | 0.66**  (0.25, 0.85) | 0.62**  (0.28, 0.84) | 0.34  (-0.16, 0.69) | 0.34  (-0.17, 0.69) |
| **MAOD (mL·kg^-1^)** |  |  |  |  |  |
| 10TT | 0.81**  (0.55, 0.93) | 0.77**  (0.48, 0.91) | 0.72**  (0.39, 0.89) | 0.70**  (0.34, 0.88) | 0.79**  (0.50, 0.92) |
| 30TT | 0.87**  (0.67, 0.95) | 0.86**  (0.67, 0.95) | 0.83**  (0.59, 0.93) | 0.80**  (0.54, 0.92) | 0.82**  (0.58, 0.93) |
| 60TT | 0.75**  (0.44, 0.90) | 0.76**  (0.46, 0.91) | 0.83**  (0.58, 0.93) | 0.80**  (0.53, 0.92) | 0.78**  (0.50, 0.92) |
| 120TT | 0.53*  (0.08, 0.80) | 0.51*  (0.06, 0.79) | 0.62**  (0.21, 0.84) | 0.81**  (0.55, 0.93) | 0.77**  (0.47, 0.91) |
| 180TT | 0.52*  (0.06, 0.79) | 0.49*  (0.03, 0.78) | 0.52*  (0.07, 0.79) | 0.67**  (0.30, 0.87) | 0.81**  (0.56, 0.93) |
| **0.7MAP + 0.3MSP** | 0.97**  (0.91, 0.99) | 0.95**  (0.86, 0.98) | 0.89**  (0.71, 0.96) | 0.87**  (0.66, 0.95) | 0.91**  (0.77, 0.97) |

Values are the correlation coefficient r, with the standard error of estimate in first parenthesis, and confidence interval in second parenthesis. VO_2peak_, peak oxygen consumption. C, oxygen cost of cycling. W, watts. MAP, maximal aerobic power (VO_2peak_ / C). MSP, maximal sprint power. APR, anaerobic power reserve. [La^-^]_b_, blood lactate concentration in millimole⋅L^-1^ (mM). MAOD, maximal accumulated oxygen deficiency.

*p<0.05 significant correlation

** p<0.01 significant correlation

**QQ-plots from 10TT, 30TT, 60TT, 120TT and 180TT**


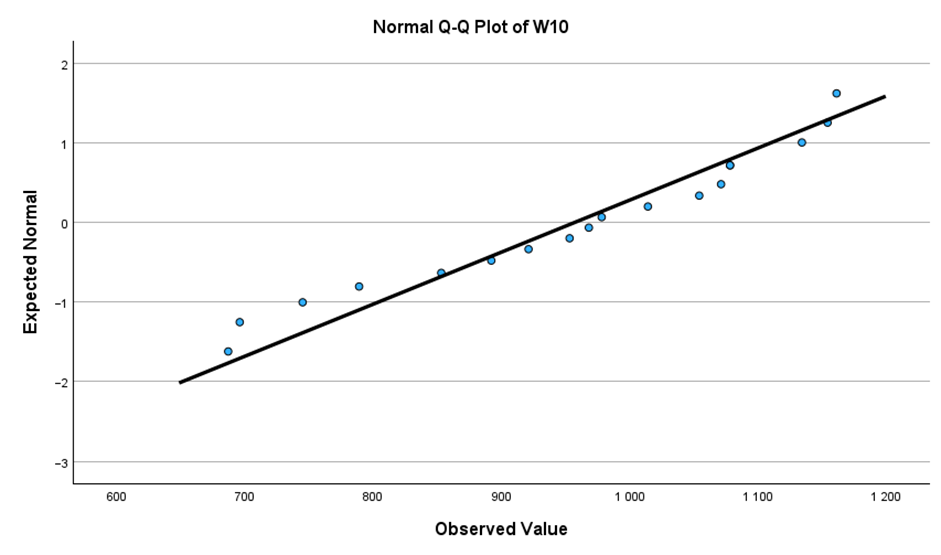


10TT


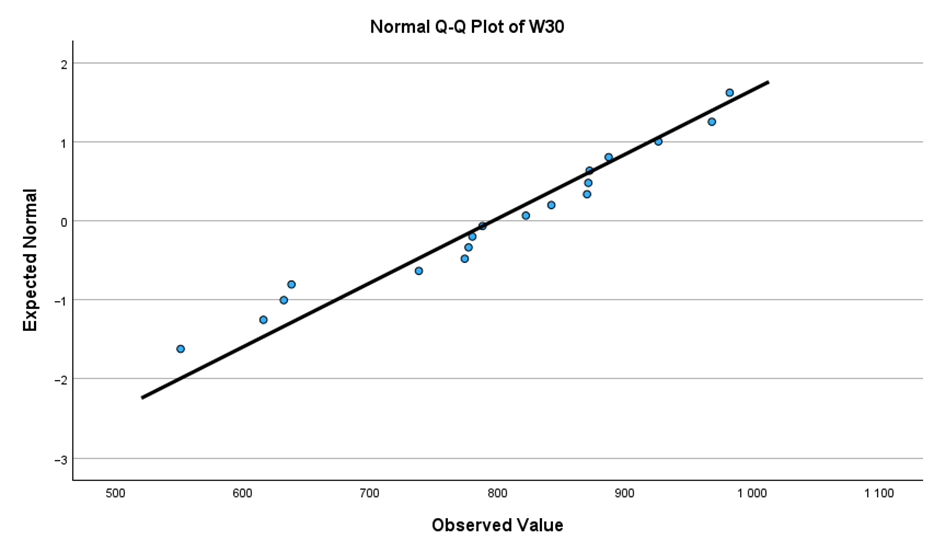


30TT


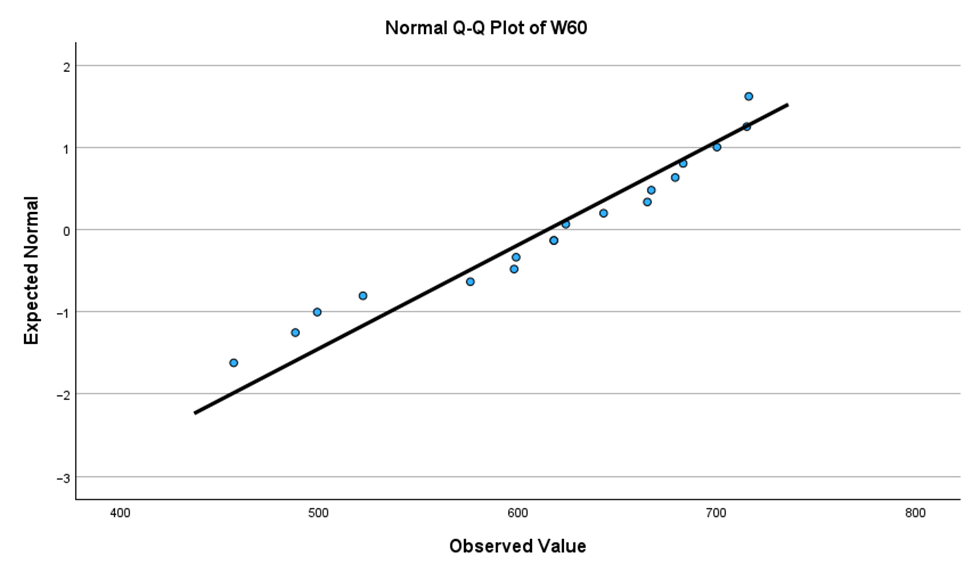


60TT


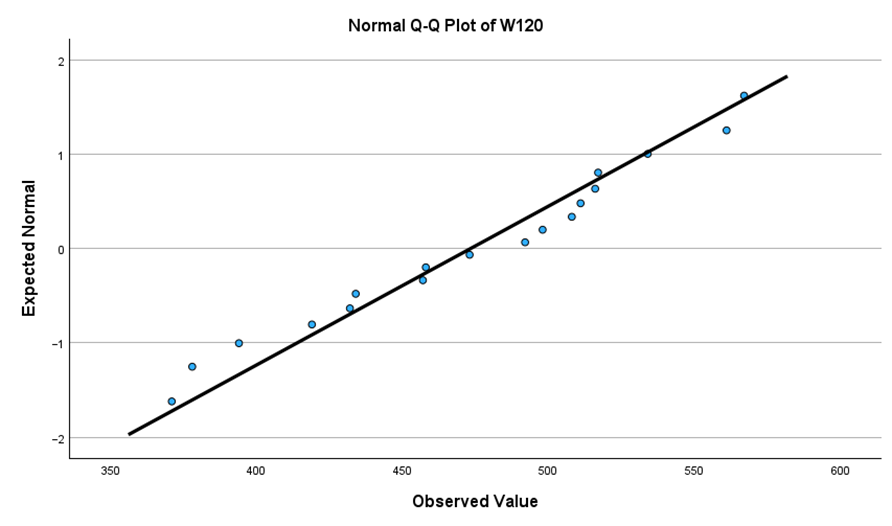


120TT


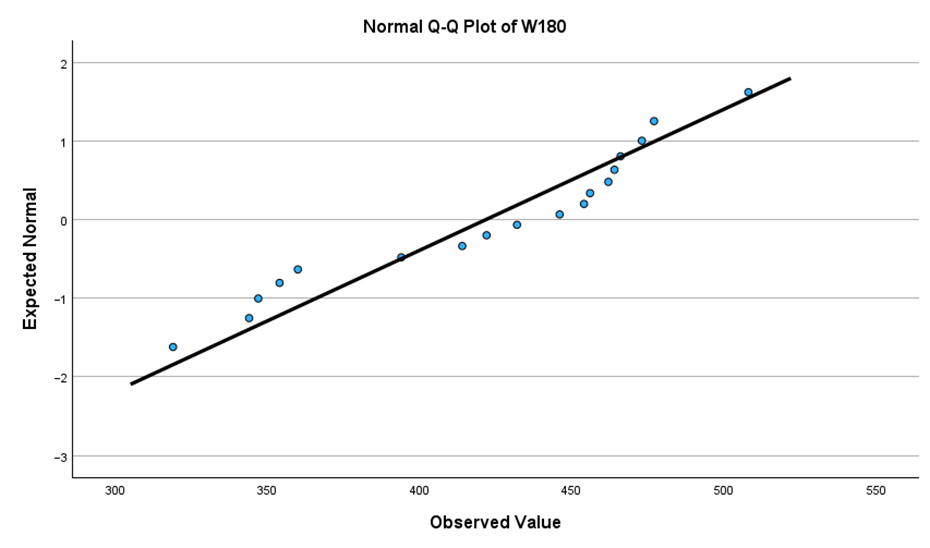


180TT
